# Recognising moulting behaviour in trilobites by examining morphology, development and preservation: Comment on Błażejowski et al. 2015

**DOI:** 10.1002/bies.201600027

**Published:** 2016-08-22

**Authors:** Harriet B. Drage, Allison C. Daley

**Affiliations:** ^1^ Department of ZoologyUniversity of OxfordOxfordUK; ^2^ Oxford University Museum of Natural HistoryOxfordUK

**Keywords:** arthropod, ecdysis, Ecdysozoa, fossil record, Trilobita, *Trimerocephalus*

## Abstract

A 365 million year‐old trilobite moult‐carcass assemblage was described by Błażejowski et al. (2015) as the oldest direct evidence of moulting in the arthropod fossil record. Unfortunately, their suppositions are insufficiently supported by the data provided. Instead, the morphology, configuration and preservational context of the highly fossiliferous locality (Kowala Quarry, Poland) suggest that the specimen consists of two overlapping, queued carcasses. The wider fossil record of moulting actually extends back 520 million years, providing an unparalleled opportunity to study behaviour, ecology and development in early animals. Taking cues from modern analogues, it is possible to quantify precise details about moulting behaviour to determine broad‐scale evolutionary trends, ontogenetic sequences and morphological selection pressures. In this review, we argue that this rich source of data has been underused in evolutionary studies, though has great potential for investigating the life history and evolution of arthropods in deep time.

## Introduction

**Figure 1:**
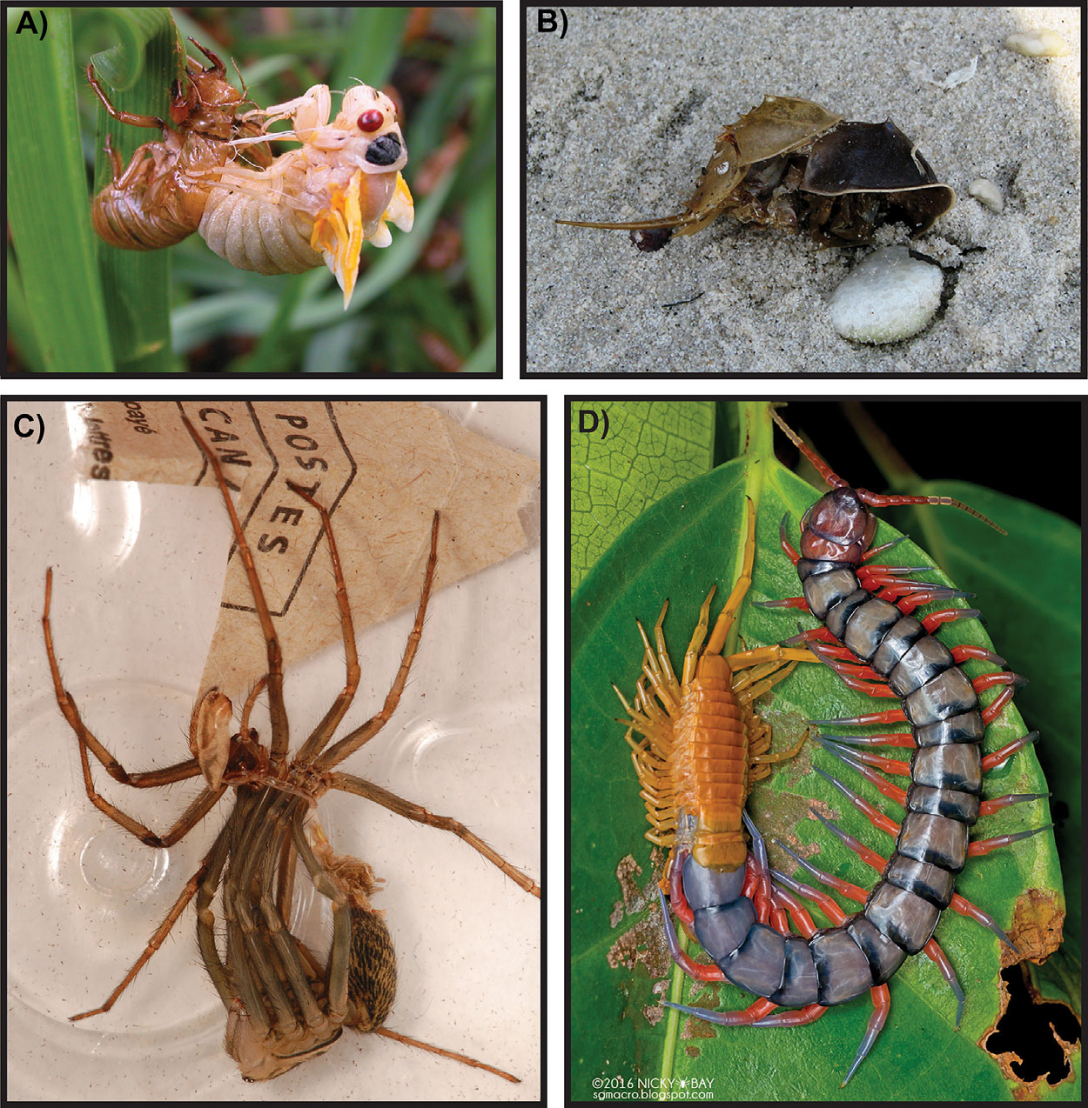
Exemplar photographs of modern arthropod moulting behaviours. **A:** Cicada moulting through the dorsal part of the abdomen (image credit: Wikimedia Commons). **B:** Horseshoe crab that died following exuviation through the anterior cephalic ecdysial gape (image credit: Wikimedia Commons). **C:** Giant house spider (*Eratigena atrica*), extracting the legs while emerging mid‐moult from the dorsal abdomen (image credit: Wikimedia Commons). **D:** Mid‐moult *Scolopendra* centipede, emerging through an anterior cephalic gape suture (image credit: Nicky Bay, http://sgmacro.blogspot.com).

The extensive fossil record of Arthropoda provides a fascinating insight into the evolution of the most diverse and abundant animal phylum, upon its appearance amongst the earliest animals 541 million years ago 1, 2, 3. Arthropod growth requires moulting of the exoskeleton (Fig. 1), a process known as ecdysis 4, 5, which is also observed in nematodes and other closely related phyla that make up the group Ecdysozoa. The exoskeleton of arthropods provides protection and structural support, but has the disadvantage of needing to be moulted in order for the animal to grow. Each growth stage in arthropods involves a moulting event, which provides the opportunity not only for development towards an adult morphology (in juveniles) and an increase in body size, but also for the regeneration and repair of a damaged exoskeleton 5. Owing to this multi‐faceted function, the mode and method of moulting can greatly influence large‐scale evolutionary trends within the arthropods; for example, the successful radiation of insects (Fig. 1A) was linked to the regulation of ecdysis‐related hormones 6. The evolutionary significance of moulting in extinct animals can also be examined because the empty moults, or exuviae, are discarded after ecdysis, providing a record of previous developmental stages, and have the potential to be fossilised. As one of the few behaviours with palaeontological evidence, moulting provides a rare opportunity to study ecology, growth and development in extinct arthropods, as recently reviewed by Daley and Drage 5.

**Figure 2:**
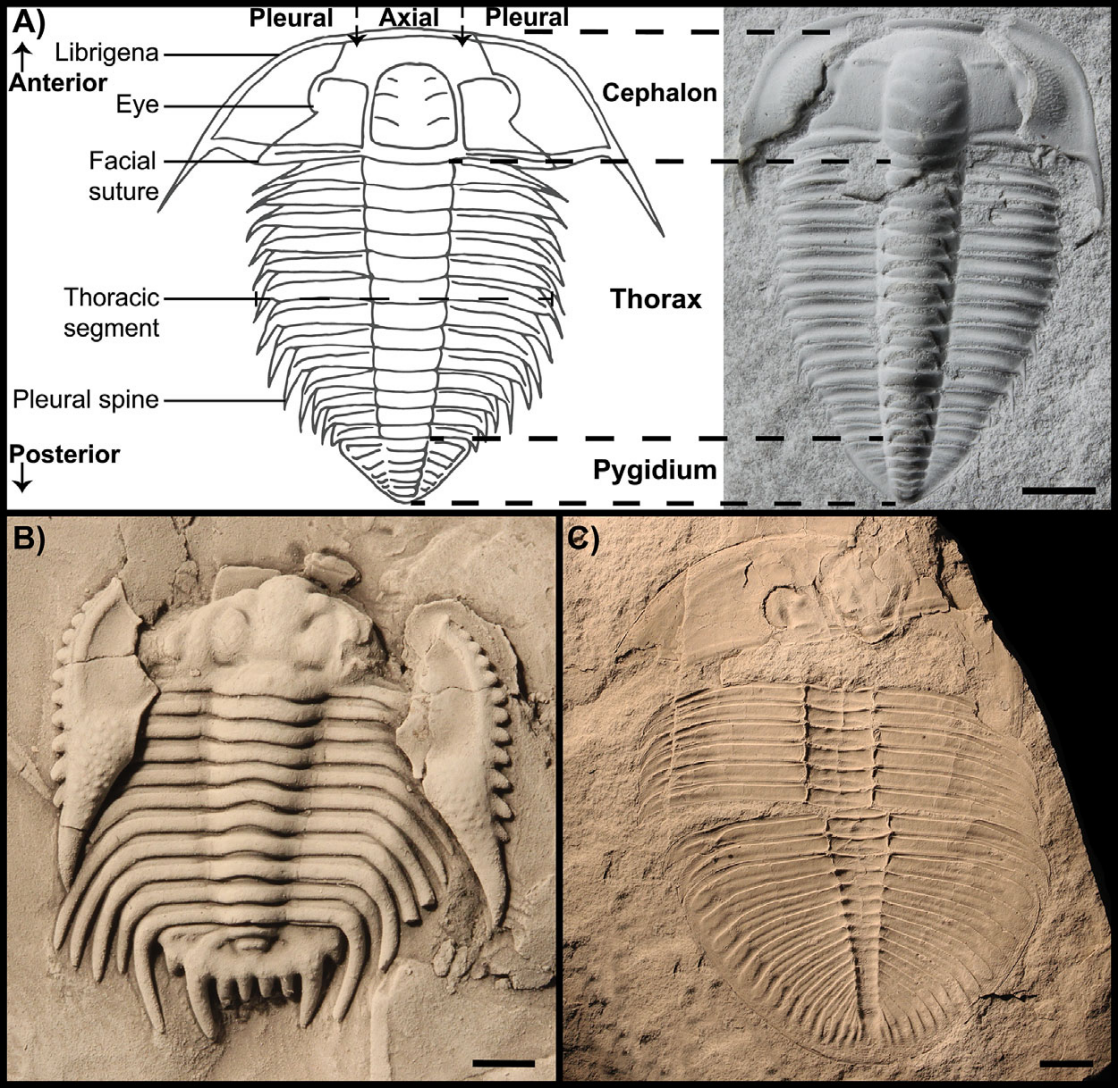
**A:** Labelled line drawing of *Olenus sp*., with the anterior and posterior directions indicated, and the cephalic, thoracic and pygidial regions also indicated on an adjacent photograph of an *Olenus truncatus* specimen (PMU, no number, 4 mm scale). **B:** *Acidaspis coronata* moult showing disarticulation and backwards displacement of the free cheeks (librigenae) (OUMNH C.17494, 5 mm scale). **C:** *Ogygiocarella debuchii* moult showing disarticulation of the cephalon and dislocation along the thorax (OUMNH B.263, 20 mm scale).

The fossil record of moulting is largely composed of exuviae, and fossils preserved during moulting are extremely rare because these events take place on a relatively rapid time scale 5. The most prolific fossil record of moulting is observed in trilobites, an extinct group of marine arthropods that were very abundant and important members of the animal ecosystem from 521 to 250 million years ago (Fig. 2). Their anatomy consists of a mineralised, segmented body with three longitudinal lobes (a central axial lobe, and left and right pleural lobes, hence the name “trilobite”), and an anterior to posterior regionalisation consisting of a head (cephalon), thorax and tail (pygidium) (Fig. 2A). Building on this basic body plan, trilobites had a diverse morphology and ecology, occupying numerous niches in marine communities. Trilobites had distinct moulting behaviours that mostly utilised the facial sutures, thus allowing the lateral and anterodorsal parts of the cephalon to be displaced (the free cheeks, or librigenae), providing an opening through which the organism could shed the smaller exoskeleton (Fig. 2B). In some trilobites, the facial sutures were fused and the animal moulted by disarticulating the entire cephalon (Fig. 2C). With either scenario, the distinct configuration remaining as empty exuviae allows us to identify specimens as moults with relative ease. For other moulting arthropods with a known fossil record, such as decapods, scorpions, and eurypterids (sea scorpions), distinguishing between exuviae and carcasses is not as easy because ecdysial suture lines are less distinct and the moult assemblages are not as recognisable 5. Trilobites, therefore, represent one of the best model taxa for studying the evolution of moulting, growth and development in extinct arthropods.

**Figure 3:**
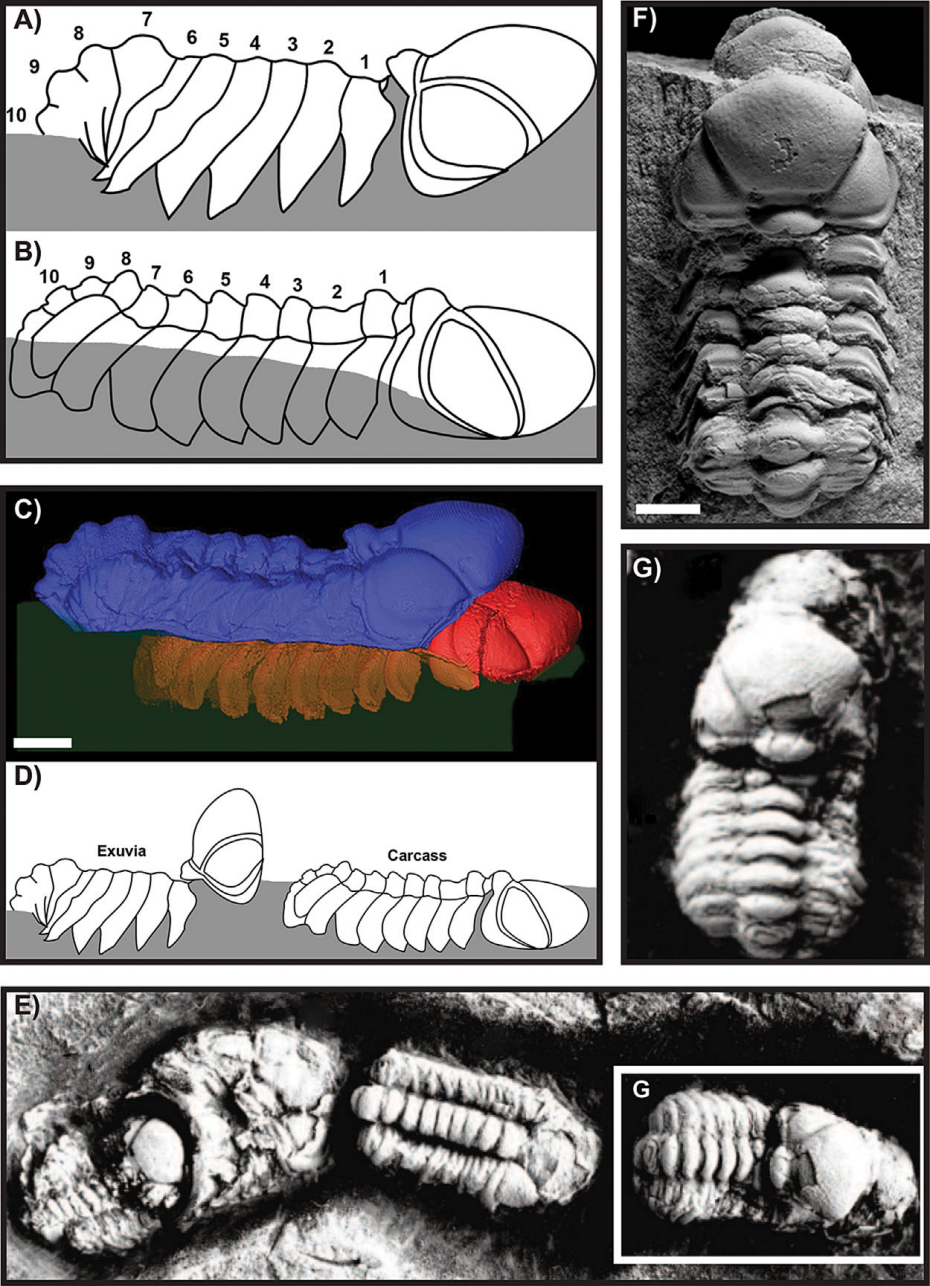
*Trimerocephalus* specimens from the Early Famennian (Late Devonian), Holy Cross Mountains, central Poland. **A:** line drawing produced from the CT scan of the *Trimerocephalus chopini* ‘moult’ figured by Błażejowski et al. 9. **B:** Line drawing from the CT scan of the *T. chopini* ‘carcass’. Numbering in A and B indicates 10 thoracic segments in each, particularly visible when using the appended CT scan video provided by Błażejowski et al. 9. **C:** Original lateral‐view CT scan of the *T. chopini* specimen from Błażejowski et al. (Ref. 9, Fig. 2B, cc‐by licensed), illustrating the relative positioning of their putative ‘carcass’ and ‘moult’. Measures of the lateral and dorsal lengths of the cephala, and of the individual thoracic segment lengths show no appreciable difference in size between the two individuals. Scale bar 2 cm, as given in Błażejowski et al. 9. **D:** In comparison to C, the positioning of the labelled ‘carcass’ and ‘moult’ suggested for a true moult‐carcass assemblage 17. **E:** One of several *Trimerocephalus* ‘queues’ of overlapping trilobite carcasses from the Holy Cross Mountains, adapted from Plate 2, Fig. 4 of Radwański et al. 19. The white box shows the contextual positioning of G. No scale bar provided in Radwański et al. 19. **F:** Dorsal‐view photograph of the *T. chopini* suggested moult‐carcass assemblage from Błażejowski et al. (Ref. 9, Fig. 2A, cc‐by licensed). Scale bar 2 cm, as given in Błażejowski et al. 9. **G:** Dorsal‐view close‐up of two overlapping *Trimerocephalus* carcasses, adapted from Radwański et al. (Ref. 19, Plate 2, Fig. 4A and B) and shown in situ in E. Shown free of context in comparison to F, the two specimens appear extremely similar. No scale bar provided in Radwański et al. 19.

Trilobites have a rich fossil record of carcasses and exuviae, which has provided a wealth of information on developmental stages, behaviour and interactions with the environment (e.g. Refs. 5, 7, 8). Trilobite specimens preserved in the short period immediately post‐moulting are extremely rare. However, a recent paper by Błażejowski et al. 9 described an Upper Devonian fossil, approximately 365 million years old from the Kowala Quarry in the Holy Cross Mountains of central Poland, that consists of two stacked exoskeletons of the trilobite *Trimerocephalus chopini*, which they interpret as in situ preservation of an individual having just emerged from its moulted exuvia, located directly above the carcass (Fig. 3C and F). The spectacular preservation of such a short‐lived event would provide important information for studying the complex interplay between ontogeny, behaviour and fossil preservation, and as such it is critical that such evidence is identified correctly in the fossil record. Unfortunately, the proposed trilobite carcass‐moult assemblage hypothesised by Błażejowski et al. 9 is not well supported, and the lines of evidence on which their interpretation is based are discussed in order below. We then place our paper in the context of the excellent fossil record of moulting in general, and focus on its potential for studying the evolution of developmental processes in deep time.

## The earliest fossil record of moulting extends back to the Cambrian

Błażejowski et al. 9 claimed that the three‐dimensional specimen of *T. chopini* from Kowala Quarry in central Poland represents the oldest direct example of a fossilised moulting event at 365 million years old. However, in‐the‐act moulting is conclusively known from a number of older fossils from the Cambrian. An exquisite specimen of *Marrella splendens* from the middle Cambrian Burgess Shale (approximately 508 million years old) was illustrated emerging anteriorly from its exoskeleton, and therefore represents the earliest example of an arthropod preserved mid‐moult 10. Even older examples (from approximately 518 million years ago) are known for other ecdysozoans, including a loriciferan (microscopic marine sediment‐affixed animals with a protective outer layer) from the lower Cambrian Sirius Passet preserved exiting its exoskeleton 11, and a lobopodian (extinct worm‐like ecdysozoan with paired limbs) with overlapping plates from the lower Cambrian of northern Greenland that suggests it was preserved during the process of moulting 12. The early fossil record of moulting is also rich in examples of exuviae, particularly for Cambrian and Ordovician strongly biomineralised arthropods such as trilobites 5. Several specimens from this time period show clusters of exuviae preserved in close proximity, indicating synchronous mass moulting events 13, 14. This rich fossil record of moulting has allowed for quantitative analyses investigating moulting behaviour trends, and their impact on trilobite evolution 5, 15. Therefore, the Devonian specimen of *T. chopini* described by Błażejowski et al. 9 cannot be considered ‘the oldest direct evidence for a moulting episode’ (p. 1).

## Specimen position, preservation and context suggest queueing trilobite carcasses

Many arthropods have distinct exoskeletal arrangements upon moulting, which are found preserved in the fossil record due to their reinforced cuticle. These moult assemblages can usually be recognised by a number of criteria, described by Henningsmoen 16 and expanded upon in Daley and Drage 5. For example, opened gape sutures (created by the opening of suture lines or articulation points) in the exoskeleton and repeated configurations of disarticulated sclerites (individual units of the exoskeleton) are most commonly used to designate moults. Contextual information from the fossil locality (associated material, palaeoenvironment, preservational processes), and the distortion of sclerites in consideration with local preservational processes (flattening, superimposition of elements, telescoping), are important for differentiating between preserved arthropod moults and carcasses, and for distinguishing abiotic or microbial exoskeleton disarticulation from true moult assemblages. Using these criteria, moulted trilobite exoskeletons are very often recognisable in the fossil record, and can provide a great deal of behavioural and ecological information. They display several distinct moult arrangements (such as those described in Ref. 16) with clear exuvial gapes. Most frequently, trilobites created an exuvial gape by disarticulating the free cheeks (Fig. 2B), or entire cephalon (Fig. 2C), followed by the animal extracting itself from the remaining articulated sclerites by moving anteriorly 5. Unfortunately, Błażejowski et al. 9 do not factor several crucial considerations into their description of a putative moult‐carcass trilobite assemblage. In particular, the presumed exuvial gape, relative positioning of their specimen, and the context (other specimens from the locality, and preservational conditions) do not support their hypotheses.

*T. chopini* figured by Błażejowski et al. 9 (Fig. 3) shows two overlapping individuals, a situation that has been interpreted as a ‘moulted exoskeleton’ positioned dorsal and slightly posterior to the ‘carcass’. The putative moult is exposed, displaying a cephalon with slight dorsal angling, while the putative carcass was revealed in three‐dimensions by CT‐scanning. Błażejowski et al. 9 argued that this specimen shows the moult arrangement described for *Paciphacops* preserved ‘immediately following moulting’ (p. 2). They (and we) reject the idea that this assemblage represents a specimen preserved during or immediately before moulting (with the ‘carcass’ still enclosed in the soon‐to‐be moulted exoskeleton) because the two individuals are ‘separated by a layer of sediment’ (Fig. 3F) and not ‘pancaked’ (Ref. 9, p. 2), as would be expected if they were still articulated at the time of preservation. *Paciphacops* is thought to utilise infaunal moulting (following burrowing for protection from predators), which occurs through an anterior gape suture created by ventral disarticulation of the cephalon and thorax, causing the cephalon to hinge dorsally at an angle of nearly 90° (Ref. 17, Fig. 2; our Fig. 3D). Other phacopid trilobite specimens also show possible infaunal moulting with their cephala angled at more than 90° from the sediment 18. However, the specimen of *T. chopini* (Ref. 9, Fig. 2) shows a much lower angle of cephalic hinging and displacement (Fig. 3C) than these definitive moult specimens, suggesting either very minor disruption during moulting or subsequent closure of the gape suture. Both scenarios would be unlikely if the animal had been moulting infaunally, because sediment would immediately infill the empty exoskeleton after moulting 18. Even if the specimen were moulting aboveground, a larger displacement of the cephalon would still be expected, as is regularly seen in taxa that moult in this manner (Fig. 2C) 5, 16. Regardless of whether *Trimerocephalus* was moulting infaunally or not, similar minor displacements of the cephala have frequently been observed in other trilobites from the Kowala Quarry, and interpreted as ‘resulting from a diagenetic event of compaction’ (Ref. 19, p. 461), rather than moulting. This further suggests that the dorsal individual figured in Błażejowski et al. 9 is not a moult.

**Figure 4:**
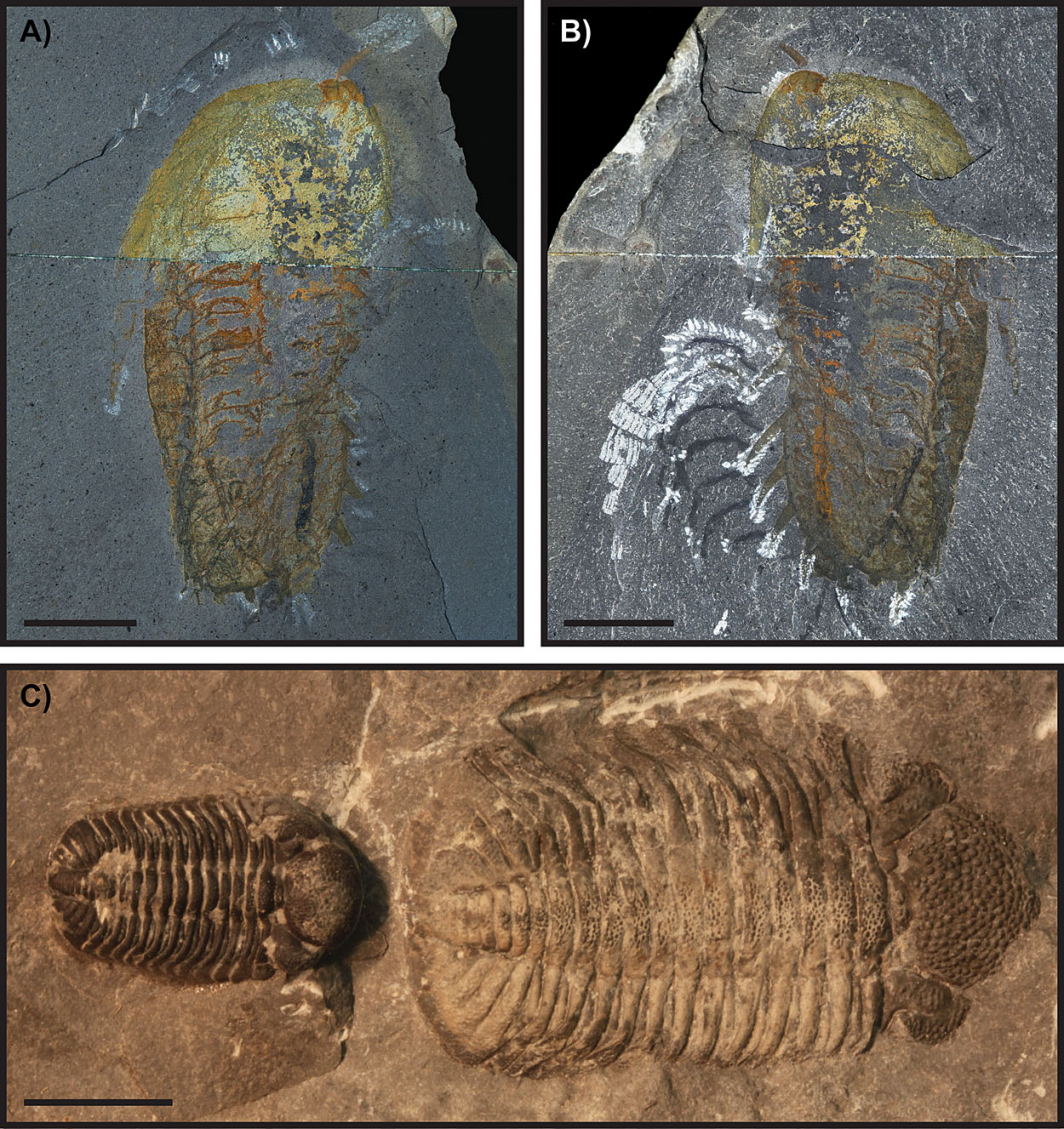
**A** and **B:** Specimen of *Olenoides serratus* described as freshly moulted 33 (USNM 57685, part and counterpart). **C:** Freshly moulted specimen of *Phacops rana* (USNM 403875, described in Refs. 31, 32). Both show wrinkling, flattening, and low relief of features; these are considered characteristic of freshly moulted trilobites and led to the original publications describing them as such. C is in contrast to an adjacent, smaller specimen considered a normal intermoult trilobite because it is darker in colour, and shows no flattening, wrinkling, or evidence of compression. Scale bars all 1 cm.

**Figure 5:**
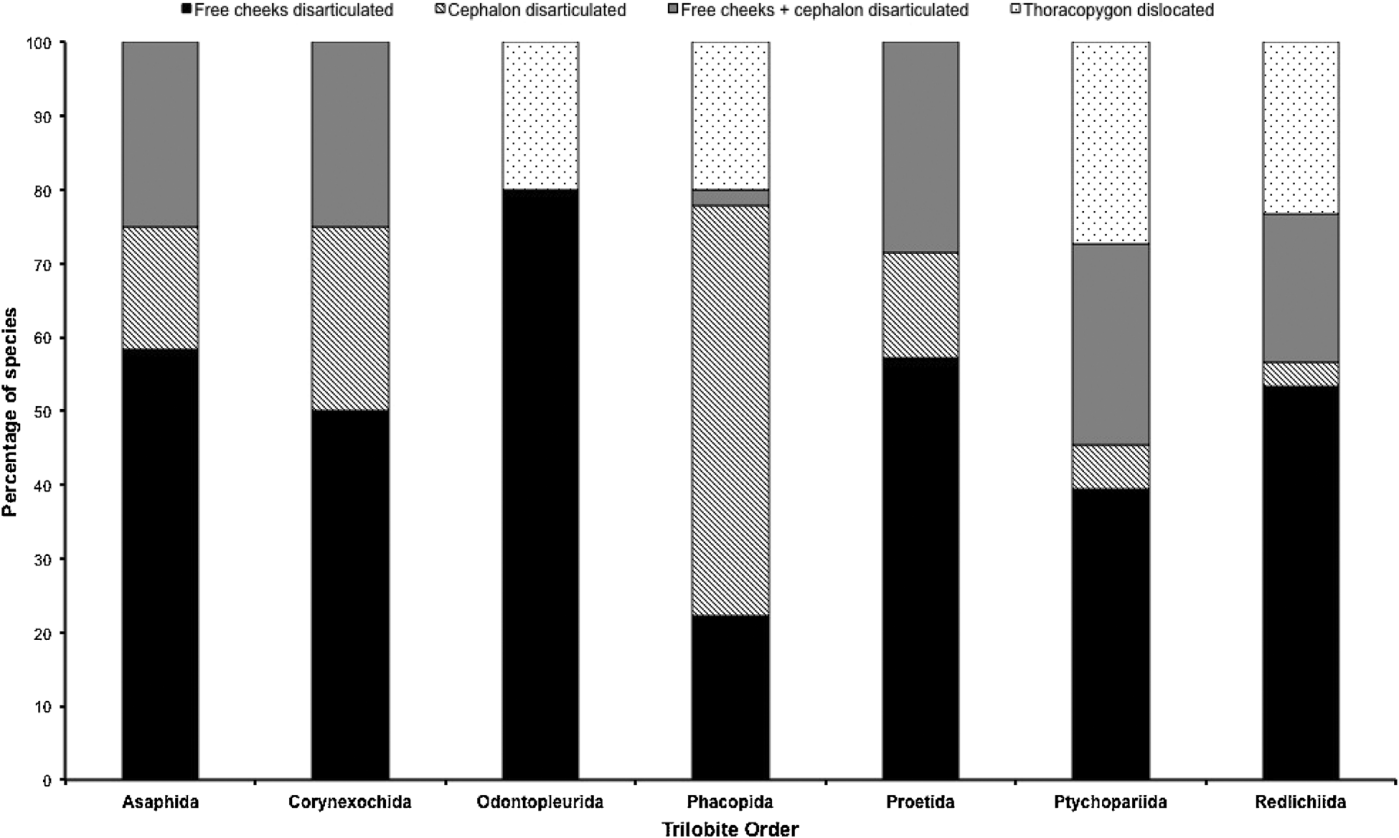
Percentage of sampled trilobite species from seven orders that show each of four different moulting behaviours (disarticulation of: free cheeks; cephalon; free cheeks + remaining cephalon; thoracic segments). Data obtained from the descriptive literature (see 5 for methodology, data normalised to 100%).

The position of the ‘carcass’ relative to the ‘moult’ raises further issues with the moult‐carcass assemblage interpretation. The ‘carcass’ component is positioned very much beneath, and only slightly forward, of the ‘moult’ (Ref. 9, Fig. 2). As mentioned earlier, this cannot represent preservation before or during moulting, because the layer of sediment separating the dorsal and ventral individuals indicates that the supposed ‘carcass’ was not enclosed in the soon‐to‐be moulted ‘exoskeleton’. To result in this position in a specimen preserved immediately after moulting, the egressing individual would have had either to detach the dorsal exoskeletal shield as a single unit and move downwards vertically, or to have exited anteriorly through a ventral gape suture under the cephalon and burrowed backwards into the sediment. Trilobites do not have sutures in the thorax, meaning that the pleural and pygidial doublure (the parts of the thorax and pygidium that extend laterally onto the ventral side) would inhibit the first of these scenarios. Some trilobites did move anteroventrally through the cephalon while exiting the old exoskeleton 16, but this would have required disarticulation of ventral sclerites (rostral plate, hypostome), and no convincing event involves backward movements within the sediment post‐moulting. The direction of exuvial movement in most trilobites is towards the anterior (Ref. 17, Fig. 3; Ref. 20, Figs. 2–6), and therefore a more convincing moult‐carcass assemblage would have the carcass preserved entirely in front of the moult on a similar depositional plane (Fig. 3D). For example, Henningsmoen 16 identified a *Hanchungolithus primitivus* specimen (Ref. 21, Plate 5, Fig. 9) possibly preserved shortly after moulting in this specific configuration (Fig. 3D) ‘again [indicating] forwards movement of the exuviating trilobite, since the larger specimen lies in front of the smaller’ (Ref. 16, p.188). The broader preservational context of the locality must also be considered. Kowala Quarry preserves aligned (‘queued’) carcasses of *Trimerocephalus* (Ref. 19, Plate 1, Fig. 5C–D, Plate 2, Figs. 3A and B, 4A and B, 6C and D) overlapping in the same way as the specimen illustrated by Błażejowski et al. 9. Radwański et al. 19 described these very similar specimens as ‘live trilobites entombed while migrating’ (p. 460), and these queues of up to nine trilobites do not show any clear evidence of moulting (e.g. disarticulated cephala) or the presence of disarticulated exuvial debris. The specimens described by Błażejowski et al. 9 more likely represent two individual aligned carcasses preserved on top of each other and separated by sediment deposition, in the queued arrangement typical of this locality 19. While this does not necessarily exclude the specimen of Błażejowski et al. 9 from also being a moult assemblage, it demands a clearer explanation of how this particular specimen differs from the other completely preserved body clusters found there. In isolation, several queued carcasses from Radwański et al. (Ref. 19, Plate 2) are indistinguishable (see our Fig. 3E and G) from the specimen described by Błażejowski et al. 9.

## Understanding moulting allows for the study of growth and development in trilobites

During development, juvenile arthropods grow in a stepwise pattern, with distinct instars separated by moulting events and accompanied by morphological change such as thoracic segment addition (anamorphic growth). Adult trilobites may have grown indeterminately, meaning they increased in size with each adult moult until death while maintaining the same morphology 22, 23. Continued moulting during adulthood is suspected because of the presence of healed injuries in adult exoskeletons, a process that only takes place during moult growth events 24. Examining moults therefore allows direct interpretation of growth and developmental patterns from the fossil record, and to date there have been numerous studies detailing variation in trilobite ontogeny (e.g. Refs. 25, 26, 27, 28). Błażejowski et al. 9 interpreted their putative moult‐carcass assemblage as a growing juvenile, based on a suggested increase in thoracic segment number. However, we find no clear evidence for the increased number of thoracic segments, and suggest that the lack of overall increase in body size argues against this being a moult assemblage.

Błażejowski et al. 9 argued that the ‘carcass’ part of their specimen displays one additional thoracic segment as compared to the supposed empty moult, and use this to support the idea of a moult‐carcass assemblage. This suggested increase in segment number may actually reflect complications in preservation of the dorsal specimen because posterior segments are obscured by pygidium enrolment. Błażejowski et al. 9 did not publish their segment number observations, but we can distinguish 10 thoracic segments in the CT‐scan reconstructions of both individuals (Fig. 3A and B). This argues against the juvenile moult assemblage interpretation. Furthermore, pygidium enrollment results from muscle contraction on death 19, and is a classic indicator of a preserved carcass rather than a moult, because complex musculature is required.

Our measurements also indicate that the overall body size and size of the individual sclerites of the ventral specimen are not significantly larger than the dorsal specimen. Trilobites grew at each moulting event 22, particularly when considered juvenile 29, as seen in the *H. primitivus* specimen figured by Dean (Ref. 21, Plate 5, Fig. 9) and suggested by Henningsmoen 16 to represent a larger carcass to the anterior of its moulted exoskeleton. While there may have been a very brief period of time immediately after moulting when inflation of body size had not yet occurred to an observable extent, the exoskeleton would necessarily be relatively soft during this freshly moulted stage, but there is little evidence of this in the Błażejowski et al. 9 specimen, as discussed in detail in the next section. A freshly moulted *Phacops* trilobite specimen found immediately adjacent to its moulted exoskeleton (Ref. 30, Plate 1, Fig. 5) clearly has a larger body size and is in the softer, post‐moult state, as evidenced by the flattened and wrinkled preservation. This specimen suggests that an increase in body size occurs almost immediately after exiting the exoskeleton, and should be observed if the specimen in Błażejowski et al. 9 really were a moult assemblage.

## Preservation of freshly moulted trilobites is characterised by wrinkling and flattening

Several trilobite carcasses (Fig. 4) have been described as preserved very shortly after a moulting event in a soft‐shelled state before the new exoskeleton mineralised and hardened 30, 31, 32, 33. In this duration of time, arthropods are vulnerable to predation. Individuals of *Phacops rana* from the same beds as trilobite exuviae clusters at the Penn Dixie shale pit, New York (Fig. 4C), ‘exhibit a distinctly thinner, pale brown to grey cuticle, which may be flattened and slightly wrinkled’ (Ref. 31, p. 96) and are found directly associated with specimens showing ‘little or no compression’ (Ref. 31, Fig. 3D, p. 91; reiterated in Ref. 32, Fig. 7E, p. 214). A single *Olenoides serratus* (Fig. 4A and B), in contrast to other individuals of the species, has low relief, conspicuous wrinkling, poorly defined structures, and soft‐part preservation of appendages and the entire alimentary canal (including midgut), the latter indicating that it is a carcass 33. *Phacops rana milleri* trilobites from the Middle Devonian Silica Shale of Ohio show the stages of post‐ecdysial cuticle hardening, freshly moulted individuals being ‘pale, thin and wrinkled’ (Ref. 30, p. 33). Cuticle thickness was quantitatively measured in thin section, and freshly moulted individuals had a thinner cuticle (25–135 µm) than intermoult specimens (300–500 µm) 30. All of these specimens show a characteristic wrinkling, flattening and thinness in the exoskeletons of freshly moulted trilobites. Wrinkles may have resulted from incomplete decompression of the new larger exoskeleton after exiting the old exoskeleton, and/or from deformation and compaction during burial in sediment, the latter of which also caused flattening of the soft exoskeleton 30. Because other preservational processes unrelated to moulting (e.g. tectonic deformation) could have caused wrinkling and/or flattening in whole assemblages of trilobite exoskeletons 34, trilobites with flattened and wrinkled exoskeletons need also to be found in close proximity to trilobites with unwrinkled, high‐relief, fully mineralised exoskeletons before they can be soundly interpreted as freshly moulted individuals. Size frequency distributions of the assemblages should also be examined to determine if differences in relief could be the result of varying preservation mechanics of differently sized trilobites.

Wrinkles in the anterior cephalon of the *T. chopini* ‘carcass’ specimen are taken by Błażejowski et al. (Ref. 9, p. 3) as evidence that the cuticle was soft as a result of recent moulting. However, these wrinkles are difficult to discern in specimen images (Ref. 9, Fig. 2E), and could alternatively be cracks in the rock, particularly given that numerous cracks are visible elsewhere in the ‘carcass’ and ‘moult’ (Ref. 9, Fig. 2C–F), and in other trilobites from this locality 19, 35, because trilobite exoskeletons are known to fracture during compression 30, 36. Wrinkles alone would not be enough to identify a freshly moulted trilobite because all other documented examples (Fig. 4) show pronounced flattening in addition to wrinkles (Ref. 30, Plate 1, Fig. 5; Ref. 31, Fig. 3D; Ref. 33, Plate 17, Fig. 3, Plate 19), owing to the greater compressibility of the softer, newly moulted exoskeleton. In contrast, the Błażejowski et al. 9 specimen shows very little difference in the height of the ‘carcass’ as compared to the ‘moult’ (Fig. 3A and B), and this lack of flattening does not support the interpretation of a freshly moulted individual.

## Modern analogues can be used to understand trilobite moulting

One of the better ways to understand the anatomy, behaviour and development of extinct animals is to compare them to modern animals with similar affinity, mode of life or environmental preferences. The use of modern analogues has shed light on a diverse set of enigmatic palaeontological evidence. For example, a comparative anatomy approach allowed for the analysis of brain morphology in a 518 million year‐old arthropod, *Fuxianhuia protensa* from the Chengjiang Biota, by comparison with the neuroanatomy of modern hermit crabs 37. Such approaches are particularly important when studying extinct animals with no direct modern relatives, as long as the modern analogue is appropriate in affinity and/or mode of life.

Błażejowski et al. 9 used horseshoe crabs as a comparison for understanding the preservation and behaviour of moulting trilobites. The horseshoe crab (Fig. 1B) is one of the most renowned ‘living fossils’, generally considered to have a morphology that has changed little in over 400 million years. In reality, horseshoe crabs (xiphosurans) have undergone striking changes to their morphology during their long evolutionary history 38. Horseshoe crabs may not be the most appropriate modern analogue for interpreting the trilobite fossil record of moulting. While trilobites and horseshoe crabs are both marine arthropods and some recent phylogenetic analyses ally them (and other Artiopoda, the extinct group containing trilobites) with the Chelicerata group to which horseshoe crabs belong 39, other analyses place trilobites with the Mandibulata, rather than the Chelicerata 1, 2, 40. Further, xiphosurans possess an anterior exuvial gape that closes after egress, making it difficult to distinguish moults from carcasses in the fossil record 5. Unlike most trilobites, horseshoe crabs have an exuvial suture that splits open along the anterolateral margin of the head, through which the animal exits, but during which no element of the head is removed 41. The resulting uncertain moult fossil record makes it difficult to study evolutionary aspects of xiphosuran moulting behaviour. Horseshoe crabs also differ from trilobites in having determinate growth, meaning that they stop growing and moulting after reaching adulthood, whereas trilobites are suggested to have continued to moult and increase in size as adults 4, 22, 23, 42, 43.

Given that trilobites have been considered closely related to Mandibulata, modern analogues may be more appropriate if drawn from within this clade, which is comprised of the Myriapoda (centipedes and millipedes), Crustacea (crabs, lobsters, shrimp, etc.) and Hexapoda (insects). Many myriapods and crustaceans show continuous growth and moulting during adulthood 4, 44, as suggested for trilobites 22, 23, 42, 43. In myriapods (Fig. 1D), moulting often proceeds by a transverse dorsal suture opening in the head to create the exuvial gape, through which the animal egresses anteriorly 45, 46. Other myriapods moult by disarticulating the entire head from the remainder of the body. The moulting behaviour and similar repeated nature of the trunk segments is therefore comparable to that of trilobites. The myriapod fossil record largely consists of millipedes, which have a robust cuticle reinforced with calcium carbonate, although most fossils are interpreted as carcasses rather than moults 47, 48. This is because in centipedes the cuticle undergoes extensive reabsorption prior to moulting and the remaining moulted exuvia is almost always consumed by the individual, which greatly reduced the chance of exuviae being preserved in the fossil record 48. Myriapods are appropriate modern analogues for studying general moulting mode, but because they are terrestrial rather than aquatic, they make poor analogues for understanding trilobite exuvial behaviour and moult preservational processes. The density difference between air and water presumably leads to divergent exuvial procedures, requiring distinct movements for terrestrial compared to aquatic moulting. Marine isopod crustaceans may be a more useful comparative group. These are extant arthropods that have a unique biphasic moulting method, in which the posterior part of the body is moulted first, followed by the anterior sclerites a few days later 49, 50, 51. Separate moulting of the body and head is reminiscent of moulting in trilobites, although in trilobites the cephalic sclerites are moulted first and the remainder of the exoskeleton is shed after. Biphasic moulting in marine isopods influences preservation of these arthropods in the palaeontological record, the majority of fossils being interpreted as moulted exuviae because they consist of posterior body regions only, without the head 50, 51. Studying how marine isopod moulting affects their fossil preservation could therefore provide insight into the taphonomy and behaviour of moulting in the extinct trilobites.

Błażejowski et al. 9 compared their proposed trilobite moult assemblage to the fossil horseshoe crab *Crenatolimulus* from the Late Jurassic Owadów‐Brzezinki Quarry in Central Poland, to examine both preservation and behaviour. The proposed wrinkles on the exoskeletons of both the horseshoe crab and the trilobite are taken as evidence for these being freshly moulted individuals that have not yet undergone post‐moulting ‘decompression’ (Ref. 9, p. 3). This is in contrast to the well‐established interpretation of wrinkles in otherwise complete horseshoe crab fossils as taphonomic artefacts resulting from sediment compaction and flattening of high relief structures 34, 41, 52. Błażejowski et al. 9 imply that the horseshoe crab specimens, and by extension the *T. chopini* trilobite specimen, are too three‐dimensional for the wrinkling seen on their exoskeletons to be from taphonomic processes. However, it has been shown that even small amounts of compression can cause taphonomic wrinkles in horseshoe crab specimens that retain high relief 34, 52. The absence of pronounced flattening in these specimens suggests that these specimens had fully hardened exoskeletons at the time of preservation, and does not support the claim of Błażejowski et al. 9 that they were freshly moulted individuals.

## Mass moulting occurs in modern arthropods and trilobites

One of the most significant social behaviours linked with moulting is the occurrence of synchronised moulting events, or ‘mass moulting’, involving a large number of individuals 31, 53, 54, 55, 56, 57. In modern arthropods, mass moulting events are sometimes triggered by external abiotic cues, as is seen in the lunar‐rhythmic moulting of the crayfish *Astacus* 53. Direct communication between individuals can also trigger synchronised moulting, either through pheromones, as seen in some colonial spiders and Hexapoda such as beetles and springtails 54, 55, 56, or through visual communication, as seen in krill 13, 57. Mass moulting reduces predation pressure on individuals during this vulnerable stage of their development 58, but the benefit is balanced by higher disease transmission rates within the population 13, 59. Synchronised moulting events are well established in modern arthropods, but identifying this behaviour in the fossil record is more difficult. Trilobites are one of the only fossil arthropod groups found in large enough numbers that we can access group behavioural information 19, 60, 61, 62, 63, 64, 65 and examine synchronised moulting events in deep time.

Gregarious behaviour in trilobites has been documented in many assemblages 19, 60, 61, 62, 63 for protection 14, moulting 31, burrowing 60, feeding 64 and reproduction 65. Unlike in modern arthropods, where behaviour can be observed directly, trilobite fossil aggregations must be considered in context to try to assess whether they are the result of gregarious behaviour or abiotic accumulations. Currents, sediment reworking, gravity and other transportation mechanisms could act to accumulate numerous individuals in one area after death, leading to a time‐averaging effect where individuals that were separated in space and time during life are combined and preserved together after death. Detailed analyses of the taphonomy, sedimentology and palaeoenvironment of the locality and size‐frequency distribution of the population have been successful in separating abiotic trilobite accumulations from actual examples of gregarious behaviour 31, 62, 65. Błażejowski et al. 9 described their Devonian *T. chopini* trilobite assemblage as preserved in close association with many juvenile individuals, and suggest that they were ‘growing in a nursery ground’ (Ref. 9, p. 4). Prior descriptions of synchronised moulting‐mating behaviour in trilobites and migration of the offspring to nursery grounds were based on firmly documented evidence of moulting, in combination with quantitative analyses of the environmental context and population structure 31, 65. These data were not described for the *Trimerocephalus* queues at the Kowala Quarry locality 9, but if documented, could allow further inferences about trilobite gregarious behaviour to be made.

## Why study moulting in trilobites?

The enormous wealth of trilobite moult fossils provides a unique opportunity to explore the development and behaviour of an ecologically important group during the early history of animal evolution. Each individual study on trilobite moulting feeds into a larger aggregation of occurrences and behavioural descriptions that can be analysed to interpret evolutionary trends for the group. This is why specimens such as that described by Błażejowski et al. 9 must be examined with a critical eye, in order to determine how appropriate they are for inclusion in future studies that examine broader trends in trilobite moulting. While most published occurrences of trilobite moulting are descriptive in nature, rare quantitative studies have given us a glimpse of the huge potential residing in this growing database of behavioural information. Brandt 15 contrasted trilobite moulting – which is actually highly variable even within a single species (e.g. Ref. 66) and lacks reabsorption of the mineralised exoskeleton before moulting – with the canalised habit and efficient reabsorption seen in modern arthropods. Preliminary survivorship analyses suggested that the ultimate extinction of trilobites may have been related to the cumulative effects of a variable and metabolically expensive moulting behaviour, revealing the evolutionary importance of ecdysial efficiency for taxonomic longevity 15, though the extent of this influence has been debated (see Ref. 67). Our own recent analyses of published occurrences quantitatively showed that trilobites exhibit a high level of variability in moulting behaviour in all orders and throughout their evolutionary history (Fig. 5, Ref. 5). These proof‐of‐concept studies show that a huge breadth of information on early arthropod development remains to be extracted and interpreted from the moult fossil record.

## Conclusions and outlook

Błażejowski et al. 9 described a specimen of *T. chopini*, from the Upper Devonian (365 million years ago) Kowala Quarry of central Poland, that they argued consisted of a freshly moulted individual preserved ventral to its recently moulted exoskeleton. This supposition is based on a suggested increase in the thoracic segment number and wrinkling of the ‘carcass’. On consideration of the preservation, morphology and arrangement of the specimen, and in comparison to other trilobite material from the locality, it is more likely that this specimen represents two separate trilobite individuals, which are queueing in a manner similar to that described for *Trimerocephalus* by Radwański et al. 19. This type of critical analysis is important when investigating the fossil record of moulting owing to the difficulty that exists in distinguishing moults from carcasses, which can be clarified by consideration of the contextual information of the fossil locality and comparison with modern analogues 5.

With every new discovery of moulting preserved in the fossil record, we are more able to discuss important aspects of the evolution of development and behaviour. The rare and exquisite preservation of caught‐in‐the‐act moulting is informative for snapshot views of ecdysial behaviour 10, 11, 12, while larger scale quantitative analyses of abundant moult assemblages (i.e. readily identifiable trilobite exuviae) allow us to link trends in moulting modes with geological time and taxonomy 5, 15. Quantitative studies on moulting in the fossil record should be further expanded, based upon their demonstrated potential to reveal information on the life histories and behaviour of extinct animals. Understanding what the fossil record can tell us about moulting and growth provides a unique deep‐time insight into the evolution and development of Arthropoda and other members of Ecdysozoa.

The authors have declared no conflicts of interest.
